# Scalability of the Heat and Current Treatment on SWCNTs to Improve their Crystallinity and Thermal and Electrical Conductivities

**DOI:** 10.1186/s11671-015-0917-0

**Published:** 2015-05-16

**Authors:** Naoyuki Matsumoto, Azusa Oshima, Shunsuke Sakurai, Motoo Yumura, Kenji Hata, Don N Futaba

**Affiliations:** Technology Research Association for Single Wall Carbon Nanotubes (TASC), Central 5, 1-1-1 Higashi, Tsukuba, Ibaraki 305-8565 Japan; National Institute of Advanced Industrial Science and Technology (AIST), Central 5, 1-1-1 Higashi, Tsukuba, Ibaraki 305-8565 Japan

**Keywords:** Single-walled carbon nanotube, Post-synthetic process, Current treatment, Heat treatment, Thermal conductivity, Electrical conductivity

## Abstract

We have investigated the scalability of our post-synthesis graphitization process for single-walled carbon nanotubes (SWCNTs), which applies heat and current to SWCNTs to improve the thermal and electrical conductivities. This investigation was performed by examining the relationship between the processing conditions and the amount of treated SWCNTs. Characterization of all cases of treated SWCNTs showed the same level of improvement of ~3 times to both the thermal and electrical conductivities and that the SWCNTs remained SWCNTs, i.e., no change in diameter or wall number. These results provided evidence that the ability to improve the crystallinity of the SWCNTs was independent of the treatment amount. Further, our results showed that an increase in SWCNT amount required increased applied current density or increased in applied temperature to achieve optimum property improvement. Finally, we found a trade-off between the current density and temperature indicating that either a high current or high temperature was required to achieve the optimum process conditions. These results demonstrated that our *heat and current* SWCNT treatment was fundamentally scalable and applied towards larger scale (i.e., gram-level or more) amounts of SWCNT.

## Background

Defects in the crystalline structure of carbon nanotubes (CNTs) represent the bottleneck for their applications. Single-walled carbon nanotubes (SWCNTs) can, in principle, possess a perfectly defined structure because the chemical structure can be completely determined by its chirality. However, the presence of crystalline defects significantly diminishes the measured properties [1]. For example, a defect-free SWCNT is predicted to possess a tensile strength of ~400 GPa, yet tensile strengths of only ~3 GPa have been experimentally observed from SWCNT fibers [2–4]. Similarly, the thermal conductivity for SWCNTs is estimated to be ~6600 W m^−1^ K^−1^ at room temperature, but the observed thermal conductivity of a SWCNT bundle (diameter, 10 nm) is only ~150 W m^−1^ K^−1^ [5, 6]. In addition, the ampacity has been theoretically estimated to be as high as 10^13^ A cm^−2^, but only 10^7^–10^9^ A cm^−2^ has been measured [7–9].

Therefore, significant effort has been invested to develop processes for CNTs to remove the defects created during the synthesis stage. Unlike purification treatments, which are aimed at the removal of catalyst, support, or carbonaceous impurities, the removal of defects represents an improvement in graphitization similar to that used for carbon fibers. Heat treatments are the most straightforward approach, and a number of works have been reported. For example, heat treatments of multi-walled carbon nanotubes (MWCNTs) at 1200–2800 °C have been reported to increase crystallinity (by Raman spectroscopy), increase purity, and improve electrical conductivity, thermal diffusivity, and mechanical compressive strength [10–13]. While the exposure of SWCNTs to high-temperature processes have shown increases in crystallinity, structural changes in terms of increased diameter and wall number have also been observed [14–18]. This structural change presents a significant problem since a wall number increase means that the SWCNTs do not remain SWCNTs. For example, the heating of SWCNTs at 1000–2400 °C has resulted in a coalescence of SWCNTs into larger diameter double-walled carbon nanotubes (DWCNTs) and MWCNTs [14, 15]. In addition, the improvement in electrical and thermal properties of SWCNTs can occur from various structural changes, such as wall number, diameter, and crystallinity, but each of these structures are affected by high-temperature treatments (1500–2000 °C) [16].

Recently, a process to remove the defects in SWCNTs without increasing the diameter and wall number was developed by applying electrical current in conjunction with heating (*heat and current*) to an “aligned SWCNT sheet.” An aligned SWCNT sheet is the result of laying the SWCNTs of a vertically aligned array (i.e., forest) flat, similar to laying down a field of corn. The result is a high-density sheet which retains the SWCNT alignment. By treating this kind of SWCNT form by the *heat and current* process, a 3.2 times increase in the Raman graphitic-to-disorder band (Raman G- to D-band) ratio, a 3.1 times increase in electrical conductivity (from 25.2 to 78.1 S cm^−1^), a 3.7 times increase in thermal conductivity (from 3.5 to 12.8 W m^−1^ K^−1^), and even a 1.7 times increase in dispersibility (from 1.7 to 2.9 mg L^−1^) were achieved [19]. While these results demonstrate the potential of our *heat and current* approach, the scalability of this treatment remains unclear. Specifically, by scalability, we mean the ability to process larger amounts of SWCNTs, achieve a similar level of improvement, and understand the scaling of the required temperature and current density.

In this research, we examined the feasibility of our *heat and current* SWCNT treatment to large-scale processing by investigating the increase in both thermal and electrical conductivities and the required optimum conditions for increased amounts of SWCNTs. We found that we could successfully treat SWCNT amounts from ~3 to ~24 mg without inducing change to the diameter or wall number by increasing the applied current density or temperature. Furthermore, we determined the optimum processing conditions (temperature and current density) that provided a similar ~3 times increase in electrical and thermal properties as well as the necessary conditions for treating larger amounts of SWCNTs. These results demonstrate that our SWCNT *heat and current* treatment is fundamentally scalable.

## Methods

### Fabrication and Treatment of Aligned SWCNT Sheet

High-purity SWCNT “forests” were synthesized using the water-assisted chemical vapor deposition (CVD) method [20, 21]. A SWCNT “forest” is an array of vertically aligned SWCNTs which are grown and self-assembled from an array of nanoparticles deposited on a substrate. In short, synthesis was performed in a 3-in. image furnace from silicon wafers, which were sequentially sputtered with Fe (1.8 nm)/Al_2_O_3_ (40 nm) as catalyst/support layer and reduced in a hydrogen ambient (H_2_/He: 90 %:10 %) at 815 °C for 15 min at a total flow of 2000 standard cubic centimeters per minute (sccm) to form catalyst nanoparticles. To grow SWCNTs, C_2_H_4_ was introduced to the growth ambient for 10 min at the rate of ~80 sccm and water vapor (200–250 ppm) using a He carrier gas (900 sccm). The forest consisted of aligned CNTs with high selectivity (99 % SWCNTs) and was very sparse (mass density, ~0.04 g/cm^3^). As the SWCNT forest was a single cohesive unit, the entire forest could be easily removed in one piece from the substrate surface with the application of light pressure to one side. In this way, the SWCNT forest was removed from the substrate, and the SWCNTs were laid flat into an aligned SWCNT sheet by sandwiching the forest between two glass slides and simultaneously applying shearing and normal forces by hand to form an “aligned SWCNT sheet” [22]. *Heat and current* treatment to the SWCNT sheets was conducted as previous reports [19] and the following section.

### Characterization

**Fig. 1:**
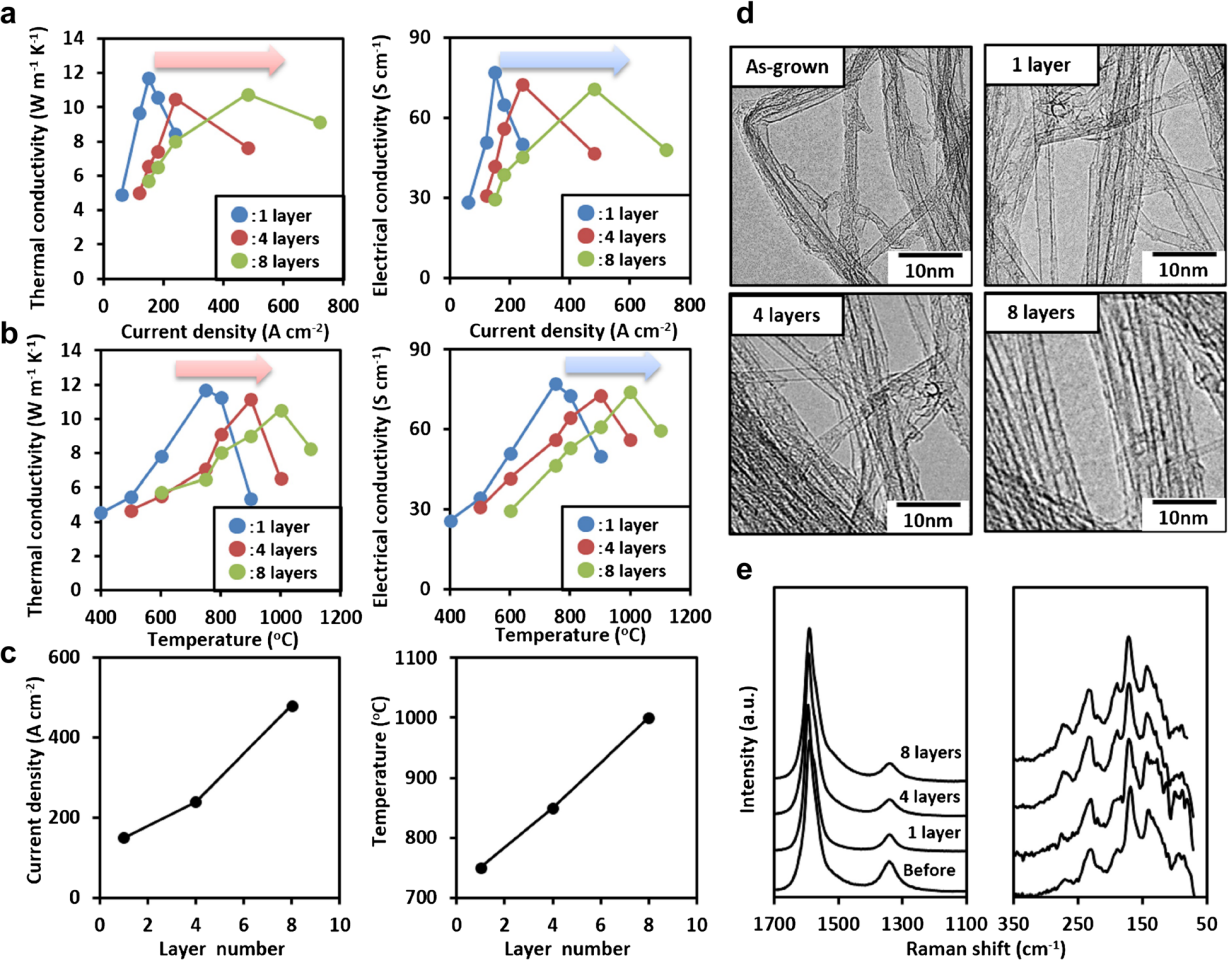
Plot of electrical and thermal conductivities after treatment as a function of (**a**) applied current density at 750 °C and (**b**) applied treatment temperature at 150 A cm^−2^. **c** Current density and temperature as functions of layer number (SWCNT amount, one layer = ~3 mg). **d** TEM images for untreated and treated (one, four, eight layers) SWCNTs and (**e**) Raman spectra for the untreated and treated SWCNTs at their respective optimum treatment conditions

The electrical and thermal properties were characterized as previous report [21, 23]. Thermal diffusivity (in plane) of SWCNT sheets was measured at room temperature in air by a thermowave analyzer (TA3, Bethel Co., Ltd). The specific heat was estimated by differential scanning calorimetry (DSC, EXSTAR X-DSC7000, Hitachi High-Tech Science Corporation). Thermal conductivity was calculated from the measured thermal diffusivity, specific heat, and sheet mass density. Electrical conductivity of the aligned SWCNT sheets was calculated from the measured sheet resistance using a four probe electrical measurement tester (Mitsubishi chemical, Loresta-EP MCP-T360) and the average cross-sectional area. The ratio of the Raman G- to D-band ratios of the SWCNT sheets was investigated by Raman spectroscopy using a Thermo-Electron Raman Spectrometer at an excitation wavelength of 532 nm. Raman characterization was performed on a 10 × 10 mm^2^ aligned SWCNT sheet on one of the parallel surfaces on several locations sampling a 100-um^2^ area. Typically, three regions were sampled across the cross-section of the SWCNT sheets, and each layer (for multiple stacked sheets) was measured to determine the representative spectra for each aligned SWCNT sheet. Our results showed that with the exception of the surfaces which contacted the graphite electrodes, the spectra across each layer (edge and middle) were quite similar. The spectra in Fig. 1 were taken from the center of the middle layer. Macro-Raman was chosen because of the large variance in spectra intensity observed for micro-sampling (up to 10 times difference), and as our SWCNT sheet samples were rather large, macro-Raman was more appropriate to average over differences caused by local differences, such as surface corrugation. The morphology and change in wall number and diameter of CNTs were evaluated by transmission electron microscopy (TEM; TOPCOM EM-002B) of dispersed SWCNTs deposited onto a standard TEM grid.

## Results and Discussion

**Fig. 2:**
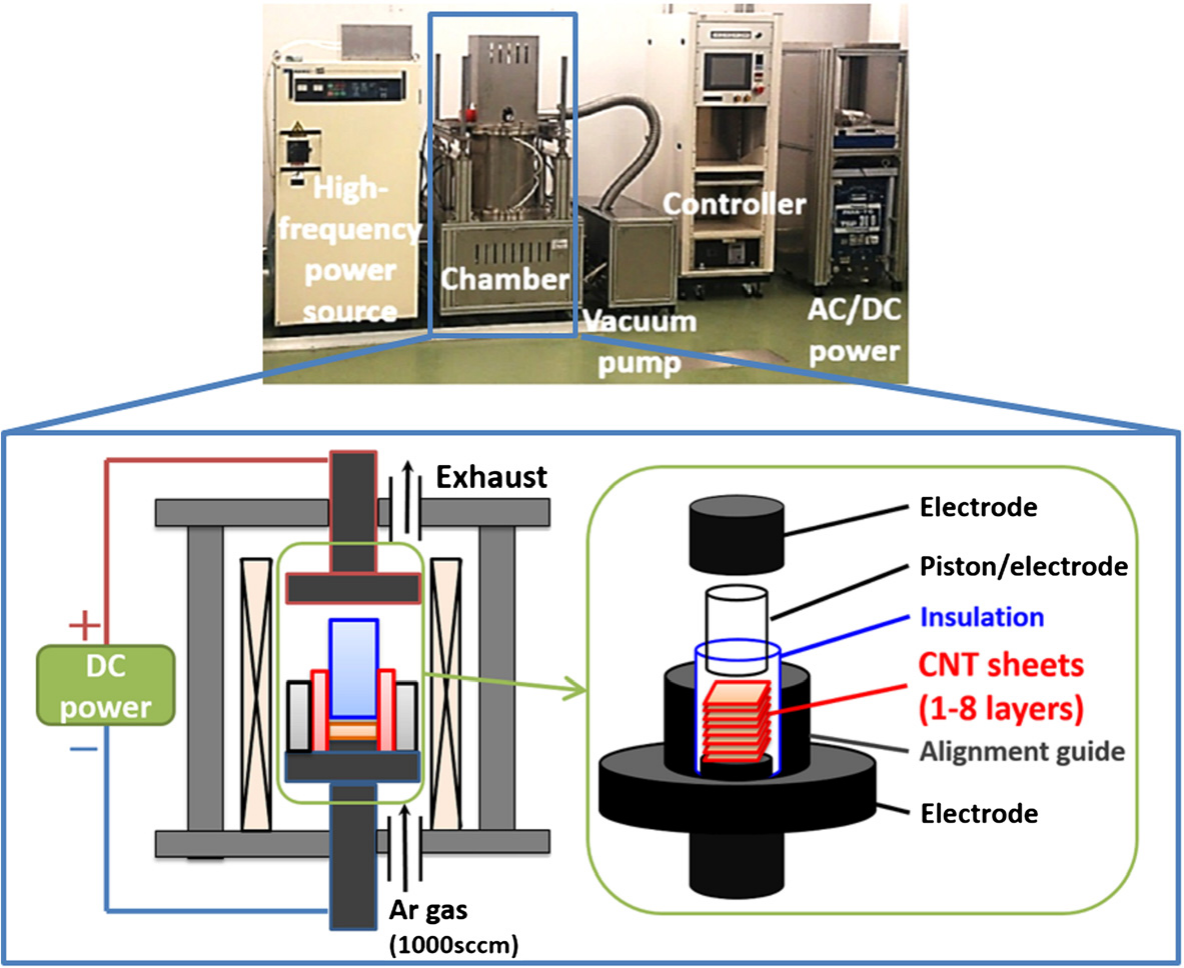
Digital photograph of the device and schematic representation of the primary interior components: Induction heater, DC power supply, gas input and exhaust, electrodes, and aligned SWCNT sheet treatment zone. Inset: zoom-in of the aligned SWCNT sheet treatment zone. The sample arrangement in the system included lower electrode, aligned SWCNT sheet(s), alignment guide, insulation between SWCNT sheets and alignment guide, piston contact, and upper electrode

We developed a custom-made treatment device that could pass large electrical currents (up to 266 A at 120 V) though the SWCNTs at high temperatures (up to 2000 °C) and in various ambient conditions (vacuum, N_2_, Ar, and H_2_ gas). A vacuum chamber was equipped with a high-frequency induction heating system and isotropic carbon electrodes that treated the aligned SWCNT sheets of 10 × 10 mm^2^ in size (~3.0 mg each) which was connected to high DC power supply (Fig. 2) [19]. These SWCNT sheets were considered “bulk” as previous methods to improve graphitization while maintaining the single-walled structure which had been limited to single SWCNTs using in situ TEM. Electrical discharge and thermal expansion were obstacles in applying high current densities at elevated high temperatures. For example, insufficient contact between the electrodes and the aligned SWCNT sheet easily resulted in electrical discharge (i.e., arcing); furthermore, if the electrodes and SWCNT sheet were first placed in contact and heated, thermal expansion of the electrodes would damage both the SWCNT sheet and electrodes. Moreover, when high electrical current was passed through the SWCNT sheet, the local temperature further increased which invoked additional thermal expansion. These issues not only prevented the progression of experiments but also made it impossible to achieve consistent and reproducible experimental results because the contact resistance between the CNTs and electrodes varied due to variations in the aligned SWCNT sheets and treatment conditions. The obstacles of thermal expansion and electrical discharge, when applying high current densities at high temperatures, were overcome by first allowing for thermal expansion by bringing the upper electrode into close proximity of the fixed bottom electrode during heating. Then, the upper electrode was brought into contact with the aligned SWCNT sheet, as determined by the measured resistance reaching 1 Ω. In this way, we could achieve a very reliable and reproducible soft contact between the SWCNT sheets.

SWCNTs (diameter, 2.9 nm; carbon purity, >99.9 %; SWCNT absolute purity, 93–95 %) within these vertically aligned forests were synthesized by the water-assisted chemical vapor deposition (Super-Growth CVD) method, which were virtually free from metal impurities that would have significantly influenced the experimental results as previously reported [20]. The high carbon purity means the near absence of non-carbon impurities, e.g., catalyst or support materials, among the SWCNTs. Despite possessing high carbon purity, the absence or low level of carbonaceous impurities is also important (i.e., absolute purity). SWCNT absolute purity was determined by outer specific surface area measurements through nitrogen adsorption isotherms using the *t*-plot formulation and was found to be >1100 m^2^/g (ideal, 1315 m^2^/g) [24]. The diameter and wall number were determined by TEM. SWCNT forests were laid flat into aligned SWCNT sheets possessing a high density and two flat parallel surfaces, which was an important feature to allow uniform and stable electrical contact with the electrodes. It should also be noted that the orientation of the SWCNTs in these sheets was virtually perpendicular to the current flow. Each aligned SWCNT sheet was 10 × 10 mm^2^ with an individual mass of ~3.0 mg and thickness of ~250 μm. In this research, we stacked one to eight layers (~3–24 mg) vertically and treated these stacks of SWCNT sheets with different temperatures and current densities and characterized the thermal and electrical conductivities. In this way, we could evaluate the optimum process conditions for each amount of SWCNTs, improvement in the SWCNT properties, and importantly, the scaling of optimum conditions with SWCNT amount.

First, we determined the optimum conditions for ~3 mg of SWCNTs (one SWCNT sheet) based on the improvement to the electrical and thermal conductivities. The required conditions were 150 A cm^−2^ at a temperature of 750 °C in an Ar ambient [19]. Based on this starting point, we investigated how the optimum conditions, based on property improvement, changed for a series of SWCNT samples (aligned sheets) which differed only by the amount (~3 to ~24 mg), i.e., one to eight layers of stacked sheets (Fig. 1a,b). For each member in this series, at a fixed temperature of 750 °C, an increase in the applied current density led to an increase in both the electrical and thermal conductivities. Importantly, in terms of the optimum level of property improvement, all members of differing amounts of SWCNTs exhibited a similar ~3 times improvement in thermal and electrical conductivities. This demonstrated that we could treat increased amounts of SWCNTs and still achieve the same level of improvement in properties by this process. However, as the amount of SWCNTs increased, the required current density also increased from ~150 A cm^−2^ for ~3 mg (one layer) to ~480 A cm^−2^ for ~24 mg (eight layers). Similarly, for each member, when holding the applied current density fixed at 150 A cm^−2^, both the electrical and thermal conductivities showed improvement with increased temperature. Again, for each case, the optimum level was ~3 times which further demonstrated the scalability of this process. These results showed that the level of property improvement did not depend on the amount of SWCNT material and that the required applied current density or temperature increased as the amount of SWCNTs increased (Fig. 1c). We wish to note that the flow of current with heat is vitally important to retain the SWCNT structure as unlike high-temperature annealing, electrons flowing through the SWCNTs are significantly scattered primarily at defect sites to locally displace carbon atoms. This is a well-known phenomenon in TEM as “knock-on” where carbon atoms are displaced by electrons [25]. Further, while the precise process remains unknown, we believe that as the amount of SWCNTs increases, the amount of defects proportionately increases, and as a result, the required current density (within the same process time) increases.

The SWCNTs within the treated sheets were characterized before and after treatment at their respective optimum treatment conditions (3 mg, 150 A cm^−2^, 750 °C; 12 mg, 240 A cm^−2^, 850 °C; 24 mg, 480 A cm^−2^, 1000 °C) by TEM and Raman spectroscopy. For each case, comparison of the treated to untreated SWCNTs showed an increase in straightness as observed by TEM, which is indicative of a reduction in defects (Fig. 1d). In addition, the average diameter and wall number of the untreated SWCNTs and treated SWCNTs were virtually unchanged (i.e., diameter, ~2.9 vs ~3.0 nm, respectively, and wall number, ~1.0 vs ~1.0, respectively). Macro-Raman spectroscopy (Fig. 1e) for each of the SWCNTs cases compared to the as-grown SWCNTs showed a similar increase in the Raman G- to D-band ratio from ~4.2 (as-grown) to ~13.1, ~13.7, and ~12.8 for 3, 12, and 24 mg, respectively (Fig. 1d). The increase in the Raman G- to D-band ratio was further indicative of a reduction in defects (i.e., increased graphitization), which is in agreement with the TEM results. Comparison of the radial breathing mode spectra (RBM) for the as-grown and all treated SWCNT sheets (3, 12, 24 mg) showed identical peaks and intensities further indicating no observable change to the diameter. Taken together, the TEM and Raman data showed that a similar reduction in defects without change in diameter and wall number was possible for each case, which further supported the fundamental scalability of this approach in treating increased quantities of SWCNTs.

**Fig. 3:**
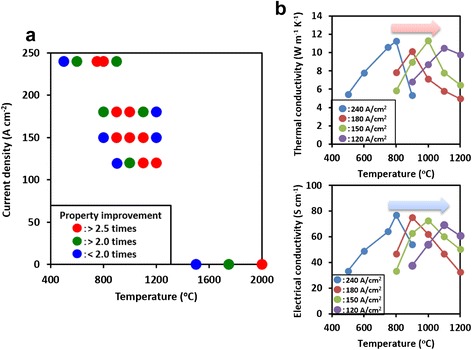
Dependence of the thermal and electrical conductivities on the applied current and temperature for a fixed SWCNT amount (four layers) and treatment time (1 min). **a** Map of the fractional increase in thermal and electrical conductivities as a function of current density and temperature. As discussed in the text, the fractional improvement is the same, which makes the simultaneous mapping of both properties possible. **b** Thermal (upper) and electrical (lower) conductivity improvement profiles for fixed current density as a function of the applied temperature

From a practical standpoint, the linear increase in required current density with SWCNT amount represents an obstacle to adopt our approach towards the large-scale (e.g., gram-level) treatment of SWCNTs, i.e., its scalability. To address the issue, we examined the dependence of the thermal and electrical conductivities on the relative levels of required current density and temperature for a fixed SWCNT amount (12 mg, four layers) and treatment time (1 min). We applied a range of temperatures (500–2000 °C) for each of the four fixed current densities (120, 150, 180, 240 A cm^−2^) and characterized the thermal and electrical conductivities and plotted them as a function of applied temperature (Fig. 3b). Each of the individual profiles, which were for a fixed current density, showed a clear peak which indicated that the optimum condition for thermal and electrical conductivity improvement was located. Importantly, the peak level was similar for these three current density profiles, indicating that more than one set of applied conditions could achieve the same level of property improvement. Interestingly, as previously observed, the fractional increase of the electrical conductivity and the thermal conductivity was nearly identical [19], i.e., the peak improvement for both properties was ~3 times. Therefore, this afforded us the opportunity to plot the fractional improvements for both electrical and thermal conductivities on a map of the temperature and current density to examine the shift in the optimum process conditions (Fig. 3a). From this plot, we observed that as the applied current density decreased, the optimum applied temperature increased nonlinearly. This means that there is a trade-off between the current density and temperature, and a requirement exists where either a high current density or high temperature is necessary to achieve the optimum process conditions.

## Conclusions

We have examined the scalability of our *heat and current* SWCNT treatment by investigating the ability to process larger amounts of SWCNTs, achieve a similar level of improvement, and understand the scaling of the required temperature and current density. Our results showed that an increase in SWCNT amount required the increase in applied current density or temperature to achieve optimum property improvement. Importantly, for all cases, the SWCNTs remained SWCNTs, i.e., no change in diameter or wall number. Finally, we found a trade-off between the current density and temperature, indicating that either a high current or high temperature was required to achieve the optimum process conditions. These results demonstrate that our *heat and current* SWCNT treatment is fundamentally scalable and has the ability to treat a large-scale amount of SWCNT simultaneously to improve both the electrical and thermal properties ~3 times.

## References

[1] Collins PG. Defects and disorder in carbon nanotubes. http://www.physics.uci.edu/~collinsp/pubs/38.Collins.pdf (2010). Accessed 25 Dec 2012.

[2] Ma W, Liu L, Yang R, Zhang T, Zhang Z, Song L (2009). Monitoring a micromechanical process in macroscale carbon nanotube films and fibers. Adv Mater.

[3] Xia Y, Zhao M, Ma Y, Ying M, Liu X, Liu P (2002). Tensile strength of single-walled carbon nanotubes with defects under hydrostatic pressure. Phys Rev B.

[4] Behabtu N, Young CC, Tsentalovich DE, Kleinerman O, Wang X, Ma AWK (2013). Multifunctional fibers of carbon nanotubes with ultrahigh conductivity. Science.

[5] Shi L, Li D, Yu C, Jang W, Kim D, Yao Z (2003). Measuring thermal and thermoelectric properties of one-dimensional nanostructures using a microfabricated device. J Heat Transfer.

[6] Grujicic M, Cao G, Roy WNA (2005). Computational analysis of the lattice contribution to thermal conductivity of single-walled carbon nanotubes. J Mater Sci.

[7] Wang X, Behabtu N, Young CC, Tsentalovich DE, Pasquali M, Kono J (2014). High-ampacity power cables of tightly-packed and aligned carbon nanotubes. Adv Func Mater.

[8] Subramaniam C, Yamada T, Kobashi K, Sekiguchi A, Futaba DN, Yumura M (2013). One hundred fold increase in current carrying capacity in a carbon nanotube-copper composite. Nature Comm.

[9] Tans SJ, Devoret H, Thess A, Smalley RE, Geerligs LJ, Dekker C (1997). Individual single-wall carbon nanotubes as quantum wires. Nature.

[10] Mattia D, Rossi MP, Kim BM, Korneva G, Bau HH, Gogotsi Y (2006). Effect of graphitization on the wettability and electrical conductivity of CVD-carbon nanotubes and films. J Phys Chem B.

[11] Zhao J, Zhang Y, Su Y, Huang X, Wei L, Kong ESW (2012). Structural improvement of CVD multi-walled carbon nanotubes by a rapid annealing process. Diamond Related Mater.

[12] Jin R, Zhou ZX, Mandrus D, Ivanov IN, Eres G, Howea JY (2007). The effect of annealing on the electrical and thermal transport properties of macroscopic bundles of long multi-wall carbon nanotubes. Physica B.

[13] Musso S, Giorcelli M, Pavese M, Bianco S, Rovere M, Tagliaferro A (2008). Improving macroscopic physical and mechanical properties of thick layers of aligned multiwall carbon nanotubes by annealing treatment. Diamond Related Mater.

[14] Yudasaka M, Kataura H, Ichihashi T, Qin LC, Kar S, Iijima S (2001). Diameter enlargement of HiPco single-wall carbon nanotubes by heat treatment. Nano Lett.

[15] Yudasaka M, Ichihashi T, Kasuya D, Kataura H, Iijima S (2003). Structure changes of single-wall carbon nanotubes and single-wall carbon nanohorns caused by heat treatment. Carbon.

[16] Matsumoto N, Oshima A, Chen G, Yudasaka M, Yumura M, Hata K (2015). Elucidating the effect of heating induced structural change on electrical and thermal property improvement of single wall carbon nanotubes. Carbon.

[17] Lopez MJ, Rubio A, Alonso JA (2004). Deformations and thermal stability of carbon nanotube ropes. IEEE Trans Nanotech.

[18] Kim P, Shi L, Majumdar A, McEuen PL (2001). Thermal transport measurements of individual multiwalled nanotubes. Phys Rev Lett.

[19] Matsumoto N, Oshima A, Yumura M, Futaba DN, Hata K (2015). Current treatment of bulk single wall carbon nanotubes to heal defects without structural change for increased electrical and thermal conductivities. Nanoscale.

[20] Hata K, Futaba DN, Mizuno K, Namai T, Yumura M, Iijima S (2004). Water-assisted highly efficient synthesis of impurity-free single-walled carbon nanotubes. Science.

[21] Chen G, Futaba DN, Sakurai S, Yumura M, Hata K (2014). Interplay of wall number and diameter on the electrical conductivity of carbon nanotube thin films. Carbon.

[22] Futaba DN, Hata K, Yamada T, Hiraoka T, Hayamizu Y, Kakudate Y (2006). Shape-engineerable and highly densely packed single-walled carbon nanotubes and their application as super-capacitor electrodes. Nature Mater.

[23] Chen G, Futaba DN, Kimura H, Sakurai S, Yumura M, Hata K (2013). The Absence of an ideal single-walled carbon nanotube forest structure for thermal and electrical conductivities. ACS Nano.

[24] Futaba DN, Goto J, Yamada T, Yasuda S, Yumura M, Hata K (2010). Outer-specific surface area as a gauge for absolute purity of single-walled carbon nanotube forests. Carbon.

[25] Warner JH, Schäffel F, Zhong G, Rümmeli MH, Büchner B, Robertson J (2009). Investigating the diameter-dependent stability of single-walled carbon nanotubes. ACS Nano.

